# Deciphering B Cell Heterogeneity and Pathogenic Mechanisms in Osteoporosis Through Single‐Cell RNA Sequencing

**DOI:** 10.1155/bmri/9979105

**Published:** 2025-12-14

**Authors:** Taolve Zhou, Jiayuan Zheng, Yujun Sun, Jionglin Wu, Wenzhou Liu, Zhenxiang Zheng, Jiajie Li, Guo Fu, Fan Zhang, Yanbo Chen, Gang Zeng, Weidong Song

**Affiliations:** ^1^ Department of Trauma Orthopedics, Foot & Ankle Surgery, Sun Yat-Sen Memorial Hospital, Sun Yat-Sen University, Guangzhou, China, sysu.edu.cn

**Keywords:** B cell heterogeneity, B cell subpopulations, immune microenvironment, LGALS9-CD45 signaling, MIF-(CD74+CXCR4) signaling, osteoporosis, scRNA-seq, single-cell RNA sequencing

## Abstract

**Background:**

Osteoporosis is characterized by reduced bone mineral density and disrupted bone microstructure, leading to an increased risk of fractures. This study aimed to investigate the role of B cell subpopulations in osteoporosis and their effects on bone metabolism using single‐cell RNA sequencing.

**Methods:**

Single‐cell RNA sequencing data from primary human femoral head tissue cells of three osteoporosis patients and one non‐osteoporosis patient were obtained from the GEO dataset (GSE169396). Data preprocessing, integration, dimensionality reduction, clustering, and annotation were conducted using the Seurat package in R. Enrichment analysis, cell trajectory analysis, and intercellular communication analysis were then applied to investigate the role of B cells and the signaling pathways within B cell subpopulations in osteoporosis.

**Results:**

We identified six distinct B cell subpopulations, and further analysis revealed a higher proportion of precursor B cells in osteoporosis patients compared to the normal group. Functional studies indicated that B cells contribute to the progression of osteoporosis through inflammatory activation and the unfolded protein response. Cell communication analysis among these B cell subpopulations demonstrated markedly enhanced intercellular signaling in osteoporosis patients relative to the normal group. Notably, two critical signaling pathways, MIF‐(CD74+CXCR4) and LGALS9‐CD45, were identified as potential key regulators driving the progression of osteoporosis.

**Conclusion:**

This study underscores the heterogeneity and functions of B cells in osteoporosis, highlighting two signaling pathways implicated in disease progression. These findings offer novel insights into osteoporosis pathogenesis and suggest potential therapeutic targets for its treatment.

## 1. Introduction

Osteoporosis (OP) is a systemic disease characterized by reduced bone mineral density (BMD) and compromised bone microstructure, leading to an increased risk of fractures. Over the past few decades, OP has been regarded as a major public health concern, particularly among the elderly [1]. It is estimated that OP‐related fractures affect one in three women and one in five men over the age of 50 [2]. With the global population aging rapidly, the prevalence of OP and related fractures is projected to increase substantially in the coming years [3].

The pathogenesis of OP is multifactorial, encompassing genetic, environmental, hormonal, and lifestyle factors [4–6]. Recently, the immune system, particularly the role of B cells, has drawn increasing attention [7]. B cells regulate immune responses by producing antibody, secreting cytokines and presenting antigens [8, 9]. Emerging evidence suggests that B cells influence bone metabolism, particularly through cytokines like receptor activator of nuclear factor‐*κ*B ligand (RANKL) and osteoprotegerin (OPG) [10]. Under normal conditions, B‐cell‐derived OPG balances bone resorption and formation by inhibiting RANKL‐mediated osteoclast activation. However, in OP, this balance is disrupted, resulting in excessive bone loss [11]. Despite these findings, the single‐cell mechanisms underlying B cell‐mediated bone metabolism require further investigation.

Single‐cell RNA sequencing (scRNA‐seq) technology offers unprecedented insights into cellular heterogeneity, enabling the identification of distinct cell subpopulations and their roles in biological processes [12]. This cutting‐edge technique holds great promise for elucidating the pathogenesis of OP and uncovering the cellular interactions in the immune microenvironment of bone tissue. In this study, we aimed to investigate the distribution and functional heterogeneity of B cell subpopulations between OP and normal samples, as well as to explore the key molecular mechanisms underlying OP.

## 2. Materials and Methods

### 2.1. Data Source and Preprocessing

The scRNA‐seq data for OP were obtained from the GSE169396 dataset in the GEO database, corresponding to the published study “Single Cell RNA Sequencing in Human Femoral Head.” This study was conducted by Central South University in Changsha, China [13]. This dataset includes primary human femoral head tissue cells (FHTCs) from three OP patients and one non‐OP control. Selection criteria for cells included (a) expressing over 300 genes, (b) genes expressed in at least three cells, and (c) mitochondrial RNA content below 20% [14]. We filtered the data with the Seurat package (version 5.1.0) in R, retaining 26,574 cells for subsequent analyses [15].

### 2.2. Data Integration

After filtering, the Seurat “NormalizeData” function was used to normalize gene expression values by scaling each gene’s expression to the total expression in each cell, followed by log‐transformation. We identified 2000 highly variable genes using the “FindVariableFeatures” function, centered them with “ScaleData,” and integrated datasets using the “RunHarmony” function. This anchor‐point integration method minimized batch effects by aligning identical cell populations across datasets into shared anchor points [16].

### 2.3. Dimensionality Reduction and Clustering

To address the high dimensionality of scRNA‐seq data, the “RunPCA” function was used for dimensionality reduction based on highly variable genes. We applied Seurat’s “FindNeighbors” and “FindClusters” functions for clustering and visualized results using uniform manifold approximation and projection (UMAP) [17, 18]. Parameters were set to dims = 1 : 20 and resolution = 0.7 for effective clustering visualization.

### 2.4. Cell Population Annotation

Cell types were annotated manually based on literature [19, 20], categorizing cells into B cells, T/NK cells, myeloid cells, red blood cells, endothelial cells, osteoclasts, and osteoblasts. Subsequent analyses focused on B cell subpopulations; detailed classification of myeloid and T/NK cell populations was not conducted.

### 2.5. Dimensionality Reduction and Clustering of B Cell Subpopulations

We further analyzed B cell subpopulations using the “RunPCA” function for dimensionality reduction and Seurat’s “FindNeighbors” and “FindClusters” functions for clustering [17, 18]. UMAP visualization was performed with parameters dims = 1 : 30 and resolution = 3.0, resulting in effective clustering of B cell subpopulations.

### 2.6. Identification of Differentially Expressed Genes and Marker Genes in B Cell Subpopulations

B cell subpopulations were annotated by combining literature review and Celltypist data [19, 21, 22]. Differentially expressed genes (DEGs) were identified using Seurat’s “FindAllMarkers” function with thresholds of |log2FoldChange| > 2 and *p* < 0.05 [23]. Subpopulation‐specific marker genes were pinpointed using the FindMarkers function.

### 2.7. Gene Ontology and Kyoto Encyclopedia of Genes and Genomes Enrichment Analysis

Gene Ontology (GO) and Kyoto Encyclopedia of Genes and Genomes (KEGG) enrichment analyses were conducted using “DAVID” and “Metascape” databases. Visualization was performed with the ggplot2 package (version 3.4.4) [24].

### 2.8. Cell Trajectory Analysis

Pseudo‐time analysis was conducted using the monocle2 package (version 2.30.0) to model dynamic transitions of B cell subpopulations during differentiation [25, 26]. We used “plot_cell_trajectory” to order cells along developmental trajectories and “plot_pseudotime_heatmap” for visualizing gene expression dynamics. Results were validated using known B cell differentiation markers.

### 2.9. Intercellular Communication Analysis

Cell–cell communication was analyzed using the CellChat package (version 1.6.1), which infers and visualizes intercellular interactions from scRNA‐seq data [27]. This tool constructs communication networks by integrating ligand, receptor, and associated molecule expression data across cell populations, providing insights into intercellular signaling.

### 2.10. Statistical Analysis

Statistical analyses were performed using R (v4.1.3; https://www.r-project.org). The Student’s *t* test was performed for group comparisons when the data were normally distributed, while the Wilcoxon test was applied for non‐normally distributed data. Statistical significance was defined as *p* < 0.05 [23].

## 3. Results

### 3.1. Single‐Cell Transcriptome Profile of OP

**Figure 1:**
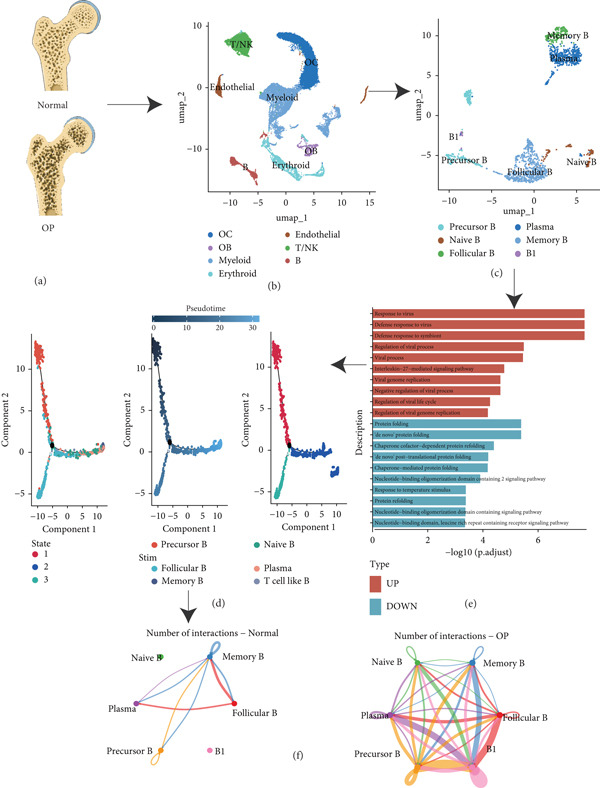
The study’s overall flowchart. (a) FHTCs of three osteoporosis patients and one non‐osteoporosis patient from the GSE169396 dataset. (b) Identification of seven cell populations based on marker gene expressions. (c) Identification of six B‐cell subpopulations based on marker gene expressions. (d) Bar chart of GO functional enrichment analysis for differential genes among B cell subpopulations. (e) Pseudotime analysis of B cell subpopulations. (f) Cell communication analysis of B‐cell subpopulations.

The study workflow is summarized in Figure 1. We analyzed four samples from the GSE169396 dataset, consisting of three OP patients and one healthy control. After quality control, 26,574 cells were retained for analysis. The “anchor point” method was used to integrate the samples and eliminate batch effects. The data were standardized, centralized, and subjected to principal component analysis (PCA), retaining the first 20 dimensions for further analysis. UMAP clustering identified 23 distinct clusters (Figure 2a). Manual annotation revealed seven cell populations: myeloid cells, T/NK cells, B cells, red blood cells, endothelial cells, osteoblasts, and osteoclasts (Figure 2b). The single‐cell transcriptome profiles of normal and OP samples are shown in Figure 2c. Figure 2d shows a single‐cell transcriptome map of different samples. Figures 2e, 2f, and 2g shows differential gene expression on seven cell populations: myeloid cells (high expression of PRTN3, AZU1, DEFA4), T/NK cells (CD3D, CD3E, TRAC), B cells (CD79A, MS4A1, CD19), red blood cells (PRDX2, GYPA, HBD), endothelial cells (VWF, CLDN5, PECAM1), osteoblasts (S100A12, MMP8, MMP9), and osteoclasts (COL1A2, CDH11, SFRP4). Compared to the control group, the OP group had a higher proportion of B cells (0.0157 in the normal group vs. 0.0592 in the OP group, Figure 2h).

Figure 2Overview of the single‐cell atlas of osteoporotic and normal samples. (a) UMAP plot depicting the clustering of single‐cell samples into 23 distinct clusters. (b) Identification of seven cell populations based on marker gene expression. (c) Comparison of UMAP clustering between normal and osteoporotic tissues. (d) UMAP clustering plot of four samples. (e) Bubble chart showing the expression of marker genes across seven cell types. (f) Heatmap showing the differential gene expression of seven cell populations. (g) UMAP plot highlighting the expression patterns of marker genes in seven cell populations. (h) Proportion of cell populations in the normal and osteoporotic samples.

**Figure:**
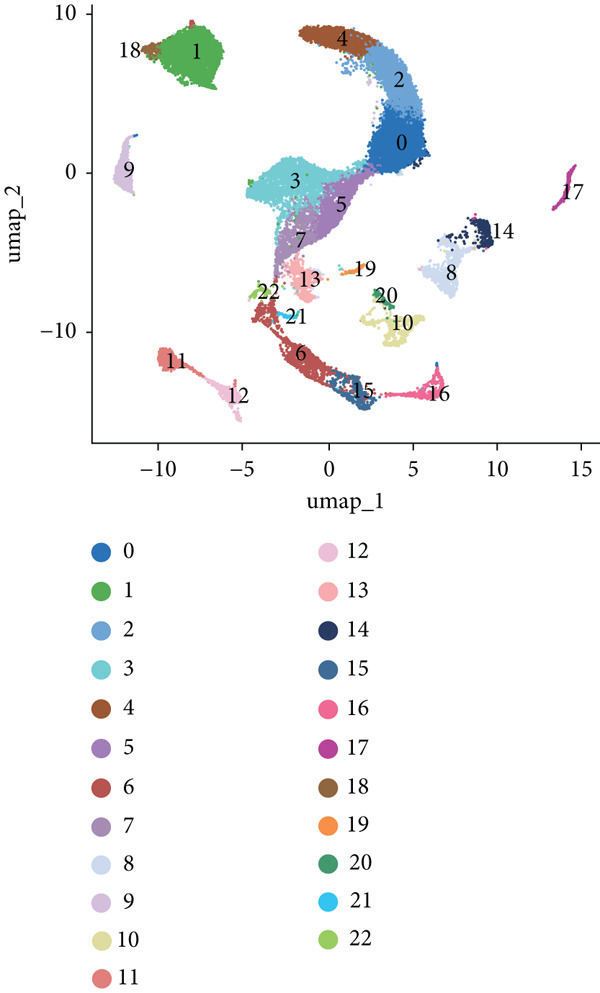
(a)

**Figure:**
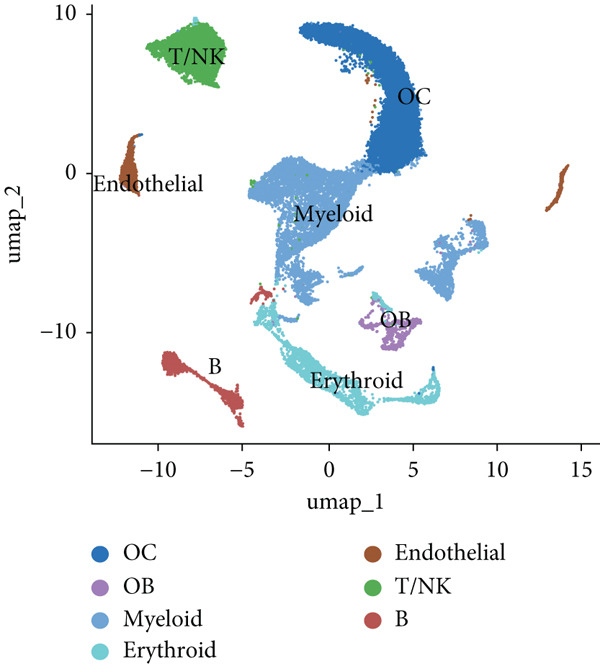
(b)

**Figure:**
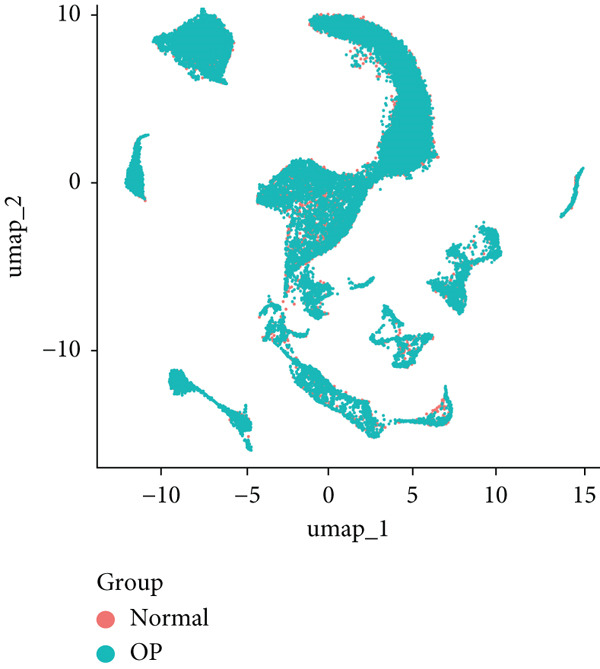
(c)

**Figure:**
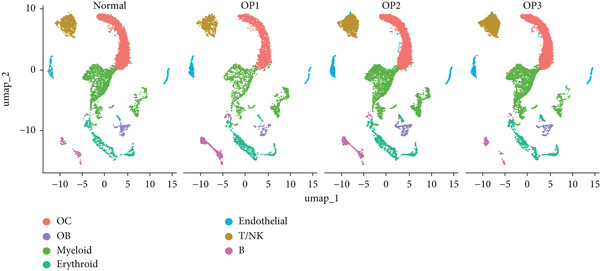
(d)

**Figure:**
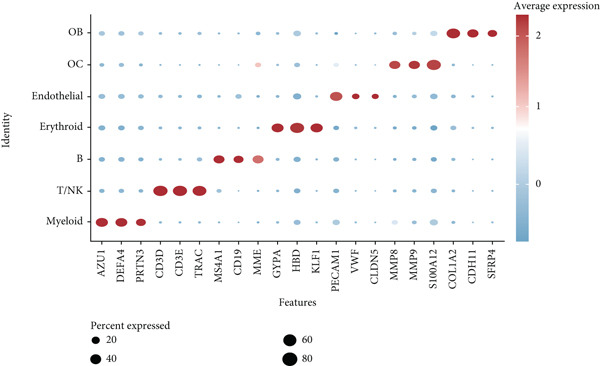
(e)

**Figure:**
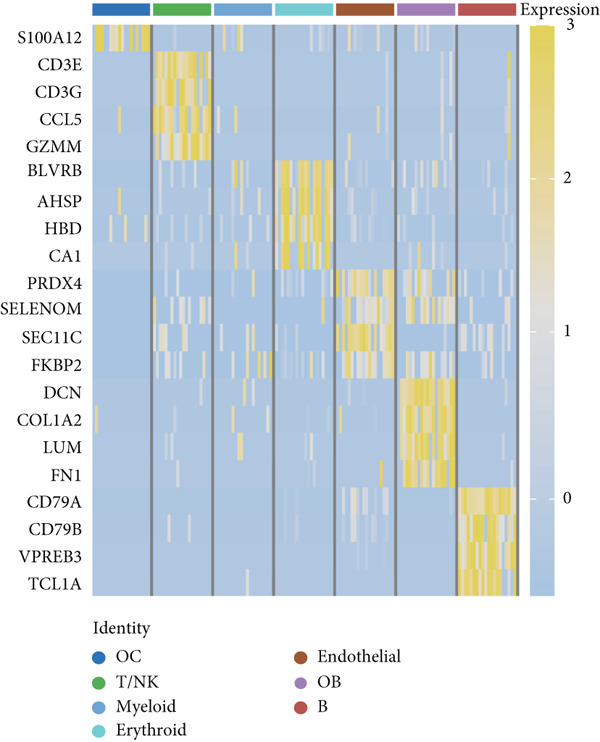
(f)

**Figure:**
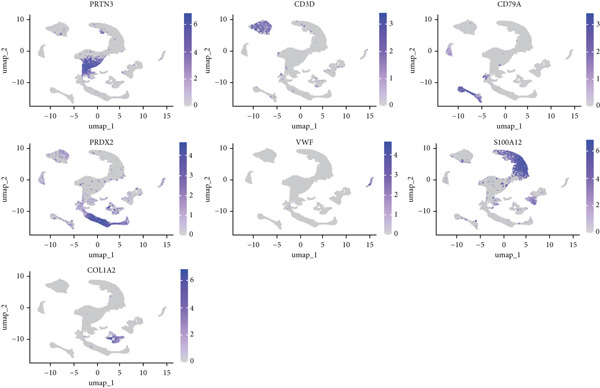
(g)

**Figure:**
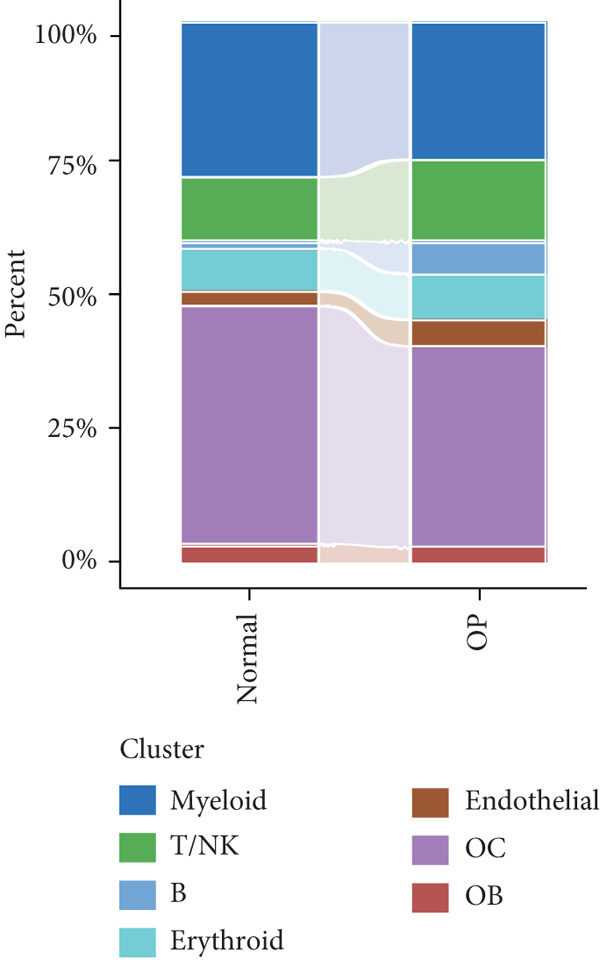
(h)

### 3.2. Single‐Cell Transcriptome of B Cells

We identified 1514 B cells across all samples. Using the same integration method, we retained the first 30 principal components for PCA and UMAP clustering, which divided the B cells into 17 clusters (Figure 3a). The B cell subpopulations were annotated as follows: precursor B cells, naive B cells, follicular B cells, memory B cells, plasma cells, and B1 cells (Figure 3b). The single‐cell transcriptome profiles of normal and OP samples are shown in Figure 3c. Figure 3d shows a single‐cell transcriptome map of different samples. Figures 3e, 3f, and 3g shows differential gene expression on six cell subpopulations: precursor B cells (ASPM), naive B cells (CYP4F3), follicular B cells (ACSM3), memory B cells (TNFRSF13B), plasma cells (PLPP5), and B1 cells (CD3E). Compared with the control group, the OP group had higher proportions of precursor B cells (0.114 in the normal group vs. 0.147 in the OP group), while memory B cells (0.167 vs. 0.104), and B1 cells (0.031 vs. 0.015) were less abundant (Figure 3h).

Figure 3Single‐cell transcriptomic profile of B cells. (a) UMAP plot depicting the clustering of B cell samples into 17 distinct clusters. (b) Identification of six B cell subpopulations based on marker gene expression. (c) Comparison of UMAP clustering between normal and osteoporotic tissues. (d) UMAP clustering plot of four samples. (e) Bubble chart showing the expression of marker genes in six B cell subpopulations. (f) Heatmap showing the differential gene expression of six cell populations. (g) UMAP plot highlighting the expression patterns of marker genes in six B cell subpopulations. (h) Proportion of B cell subpopulations in the normal and osteoporotic samples.

**Figure:**
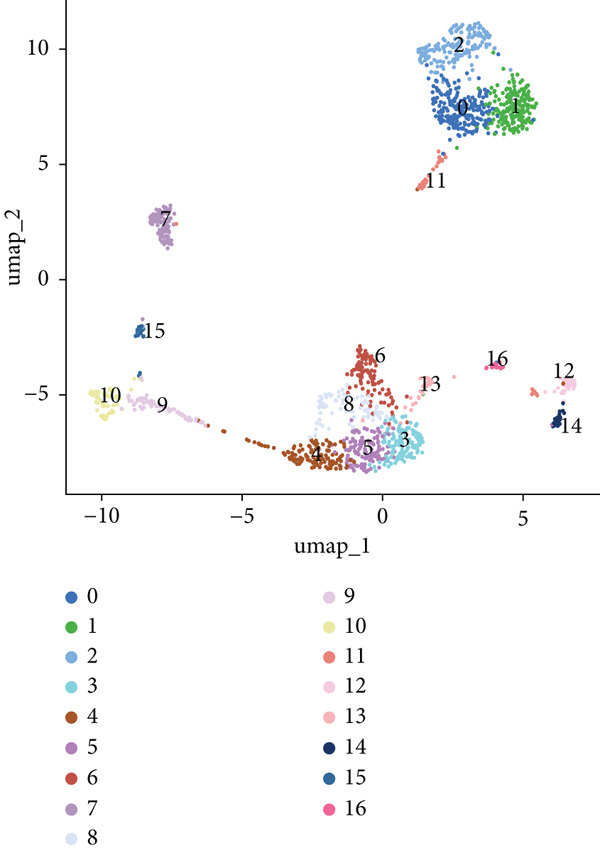
(a)

**Figure:**
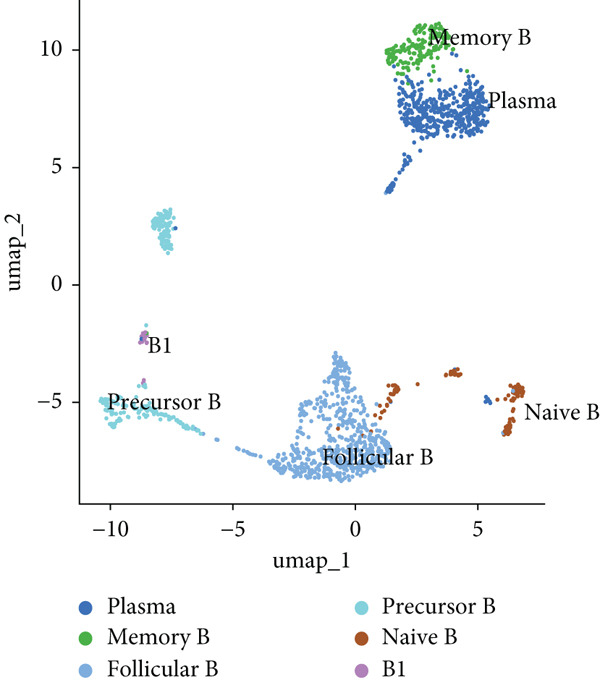
(b)

**Figure:**
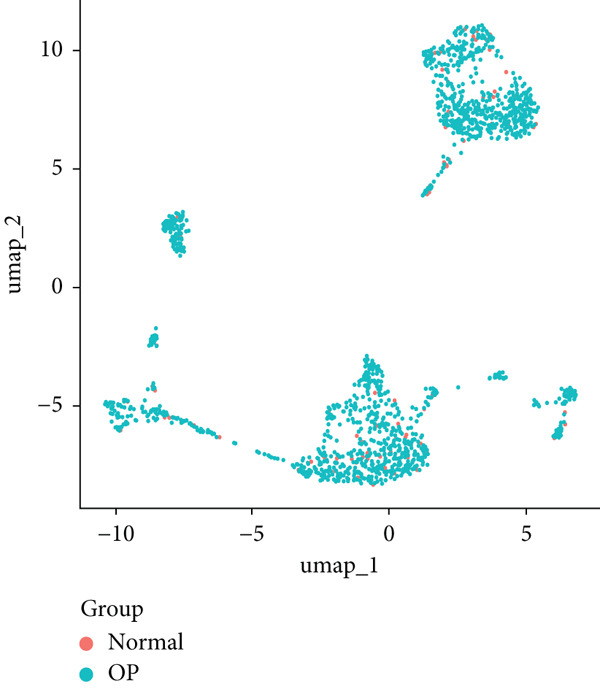
(c)

**Figure:**
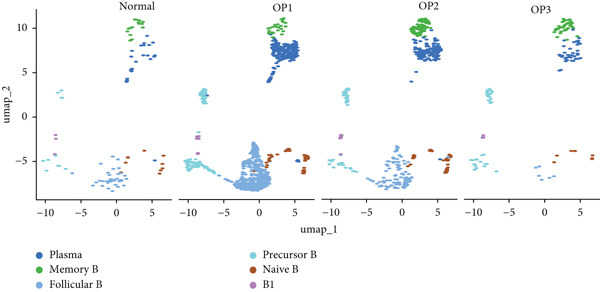
(d)

**Figure:**
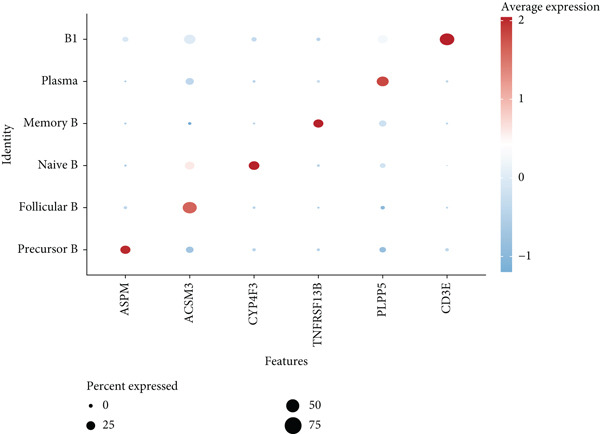
(e)

**Figure:**
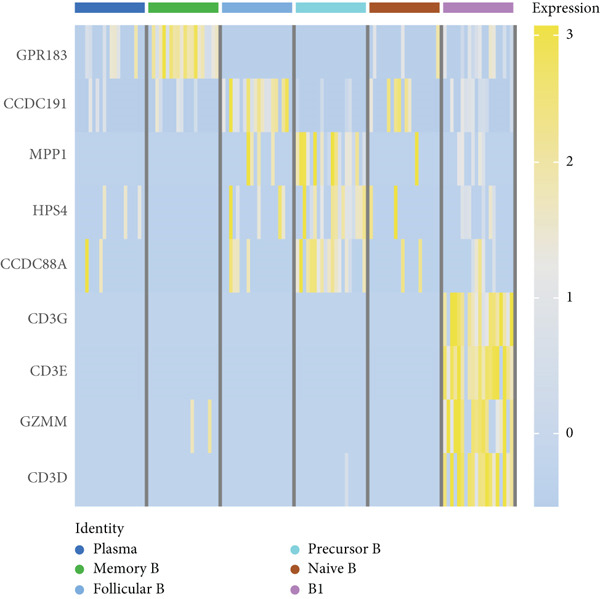
(f)

**Figure:**
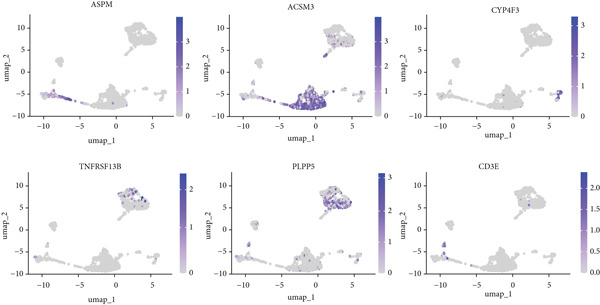
(g)

**Figure:**
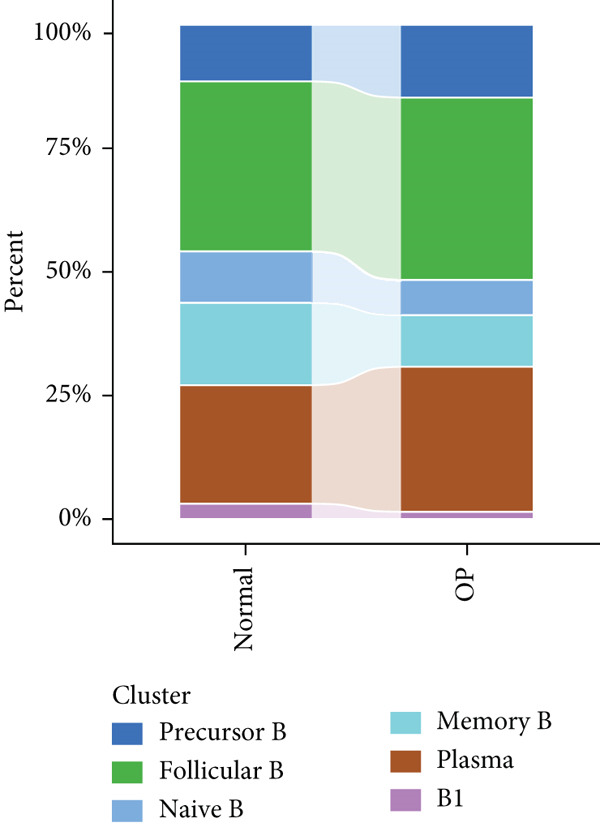
(h)

### 3.3. Functional Enrichment of B Cells

To explore the biological functions of differentially expressed genes in B cells, we conducted GO enrichment analysis comparing B cells with others. Biological processes were significantly associated with ribosome biogenesis, RNA metabolism, and tRNA processing; cellular components included the mitochondrial matrix and ribosomal subunits, while molecular functions involved RNA catalytic activity and methyltransferase activity (Figure 4a,b). For the intergroup analysis of B cells, viral response pathways and immune‐related processes were upregulated, while protein folding pathways were downregulated (Figure 4c). Then, we applied KEGG pathway analysis and showed that the marker genes were significantly enriched in protein processing, ribosome function, lysine degradation, and Epstein–Barr virus infection (Figure 4d). Differential analysis among B cell subpopulations revealed a greater number of marker genes in precursor B cells and B1 cells compared to other B cell subpopulations (Figure 4e), and volcano plots confirmed significant gene expression differences between groups (Figure 4f,g).

Figure 4Enrichment analysis of B cells across populations and groups. (a) GO functional enrichment map of DEGs between B cells and other cell populations. (b) GO functional enrichment map of DEGs between B cells and other cell populations across BP, CC, and MF levels. (c) GO functional enrichment bar chart of DEGs between the normal and OP groups. (d) KEGG functional enrichment map of DEGs between B cells and other cell populations. (e) Scatter plot of differential analysis among B cell subpopulations. (f) Volcano plot of intergroup differential analysis of B cells. (g) Diagonal volcano plot of intergroup differential analysis of B cells.

**Figure:**
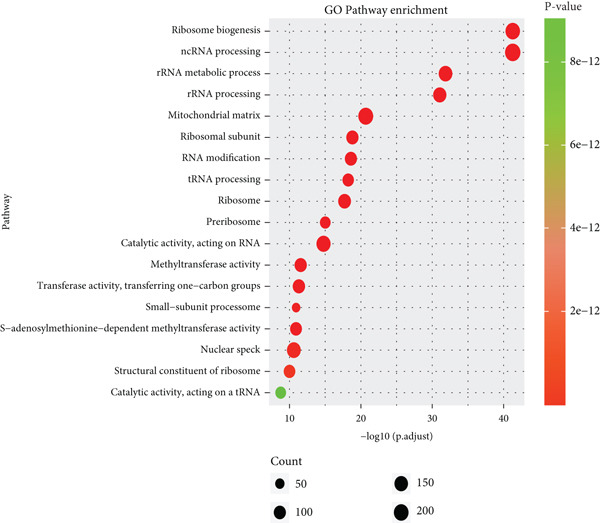
(a)

**Figure:**
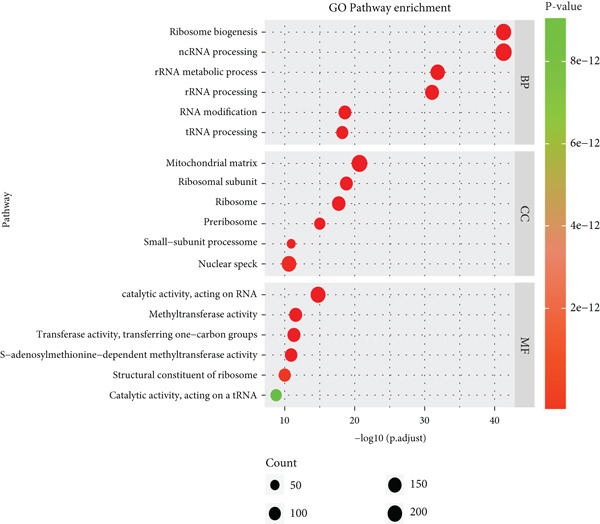
(b)

**Figure:**
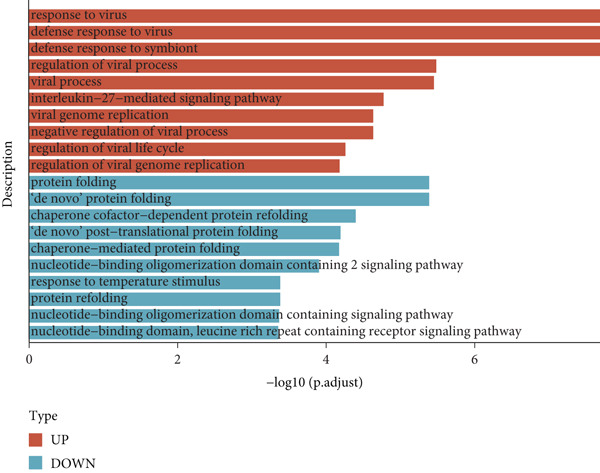
(c)

**Figure:**
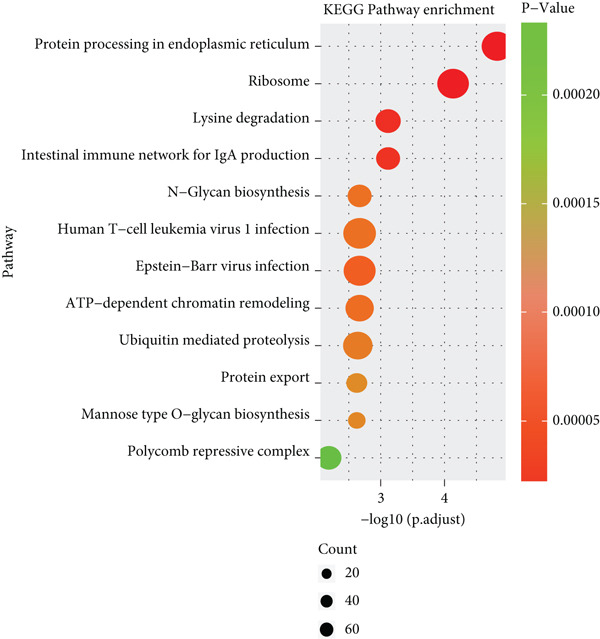
(d)

**Figure:**
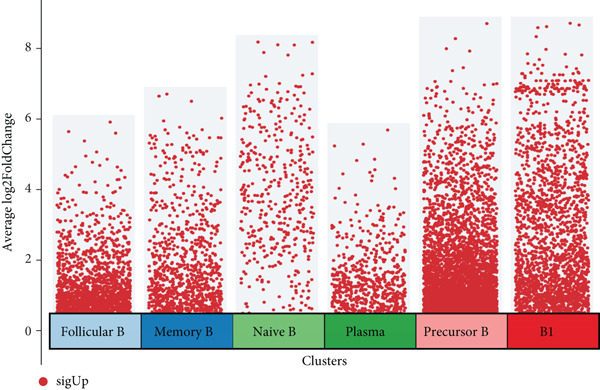
(e)

**Figure:**
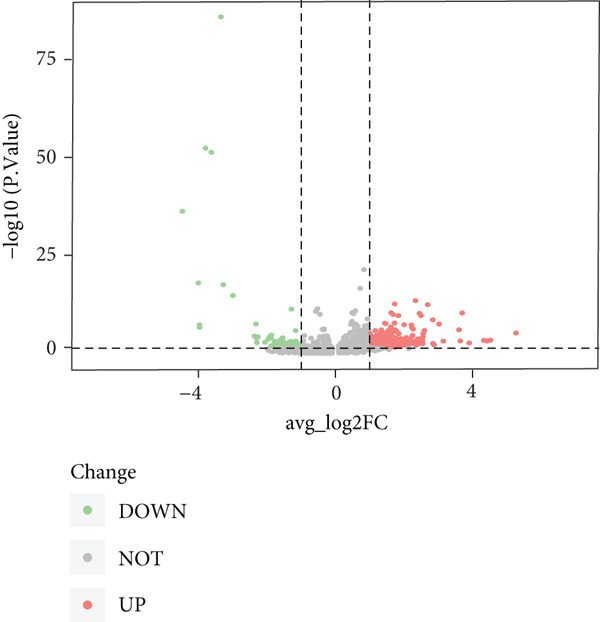
(f)

**Figure:**
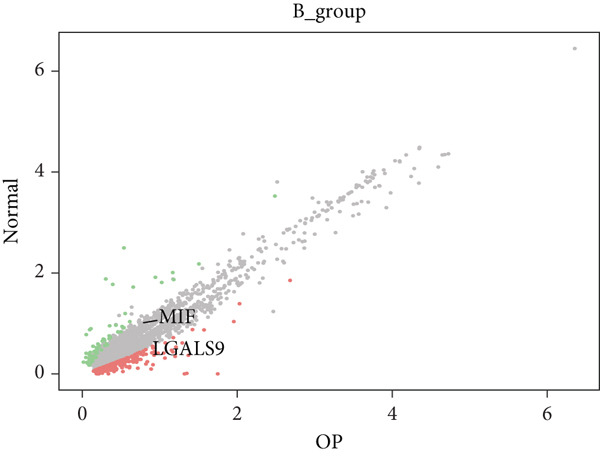
(g)

### 3.4. Pseudotime Analysis of B Cell Differentiation

Pseudotime analysis, which models the developmental trajectory of cells, showed that precursor and naive B cells are at the early stages of differentiation. They gradually differentiate into follicular B cells, which further develop into plasma and memory B cells (Figures 5a, 5b, and 5c). We divided B cells into four clusters based on gene similarity in pseudotime and performed GO enrichment analysis. Cluster 1 was associated with chromosome segregation and mitosis; Cluster 2 with lymphocyte differentiation and B cell activation; Cluster 3 with antigen receptor signaling and immune responses; and Cluster 4 with cytoplasmic translation and ribosome biogenesis (Figure 5d). Key genes such as DNTT, HMGB2, MKi67, and TUBB were expressed predominantly in early B cell stages, while GAPDH expression decreased as B cells matured but increased again in plasma and memory cells (Figure 5e).

Figure 5Pseudotime analysis of B cell subpopulations. (a) Distribution of different B cell subpopulations along the pseudotime trajectory. (b) Differentiation trajectory of B cell subpopulations in pseudotime. (c) Distribution of B cells across different pseudotime stages. (d) Heatmap showing the distribution and enrichment of DEGs along pseudotime. (e) Scatter plot illustrating gene expression changes over pseudotime.

**Figure:**
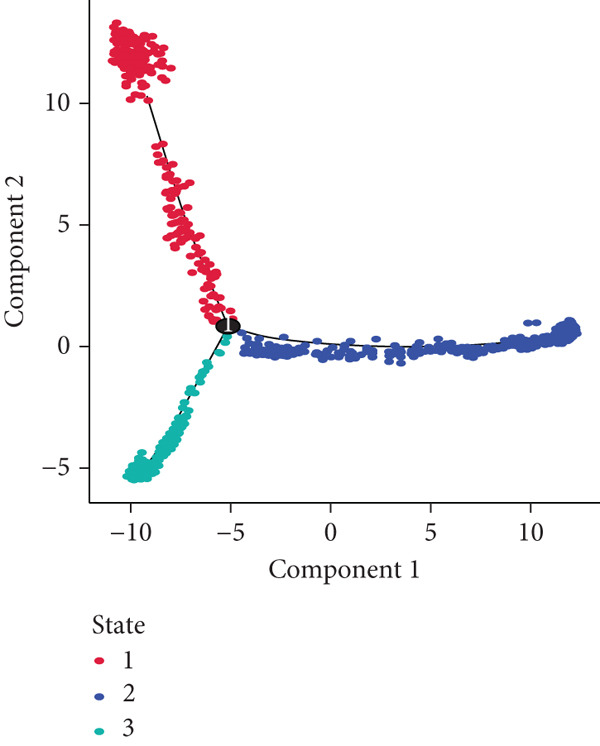
(a)

**Figure:**
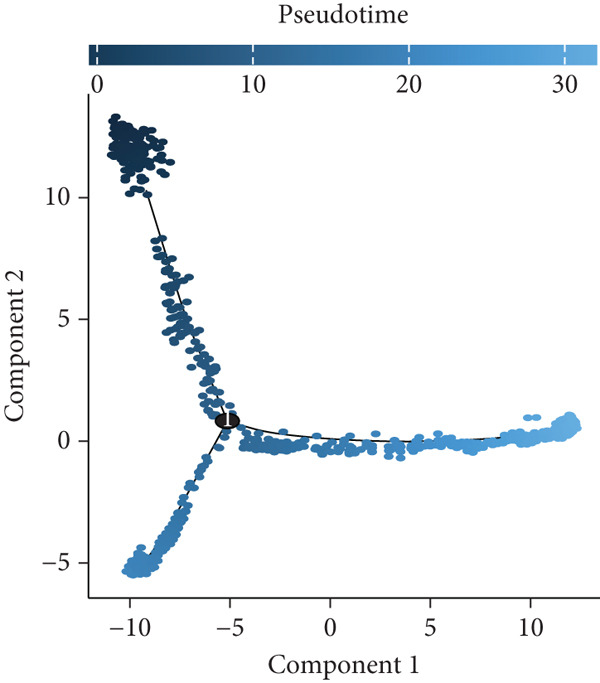
(b)

**Figure:**
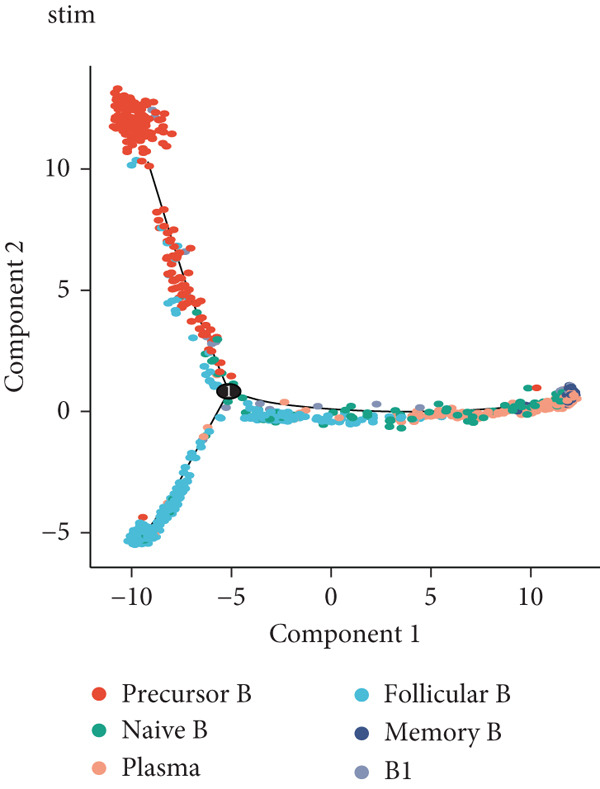
(c)

**Figure:**
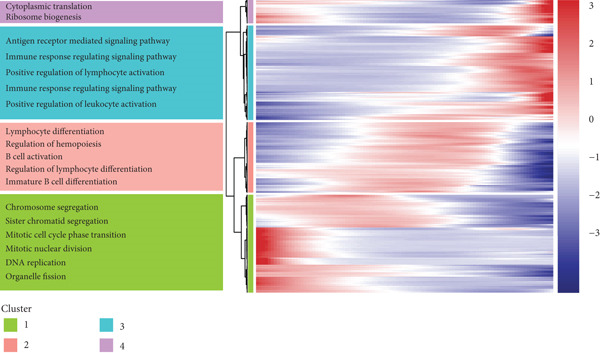
(d)

**Figure:**
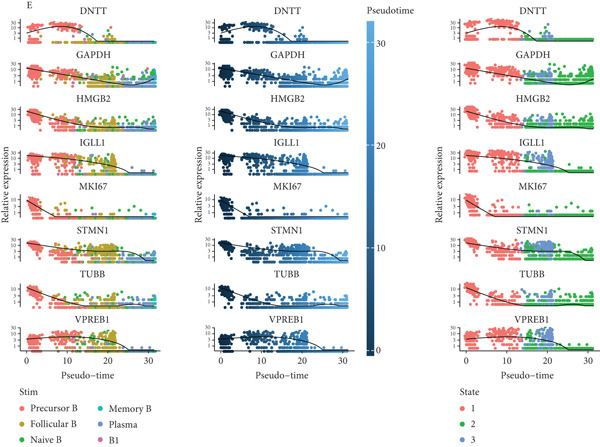
(e)

### 3.5. Communication Between B Cell Subpopulations

Figure 6a shows the differences in the number of communication pathways among B cell subpopulations (25 in the normal group vs. 180 in the OP group). Figure 6b,c further compares the intensity of communication pathways, showing a marked increase in the OP group (1.45 in the normal group vs. 7.39 in the OP group), particularly in B1 cells. Figure 6d summarizes the overall differences in pathway numbers and intensities between the two groups. Notably, Figure 6e demonstrates the specific upregulation of macrophage migration inhibitory factor (MIF)‐related pathways (*p* = 0.030) and lectin, galactoside‐binding, soluble, 9 (LGALS9 or GALECTIN‐9)‐related pathways (*p* = 6.10e − 05) in the OP group. Further analysis in Figure 6f identifies precursor B cells as key signal‐emitting cells, with prominent pathways including MIF‐(CD74+CXCR4), MIF‐(CD74+CD44), LGALS9‐CD45, and LGALS9‐CD44.

Figure 6Cell communication analysis of B cell subpopulations. (a) Network diagram of the number of interactions among B cell subpopulations in the normal group and the OP group. (b) Heatmap of the number and intensity of interactions among B cell subpopulations in the normal group and the OP group. (c) Network diagram of the number and intensity of interactions among B cell subpopulations in the normal group and the OP group. (d) Comparison of the number and intensity of B cell subpopulations communications between the normal group and the OP group. (e) Differences in the number and intensity of ligands related to signal pathways among B cell subpopulations in the normal group and the OP group. (f) Communication probabilities of signaling pathways mediated by ligand–receptor pairs between precursor B cells and other subpopulations.

**Figure:**
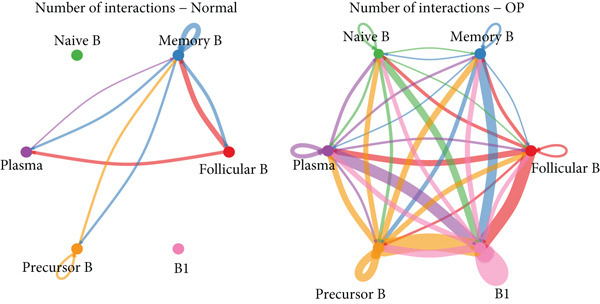
(a)

**Figure:**
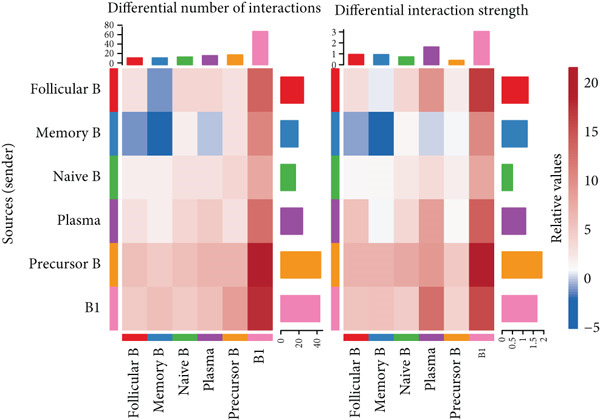
(b)

**Figure:**
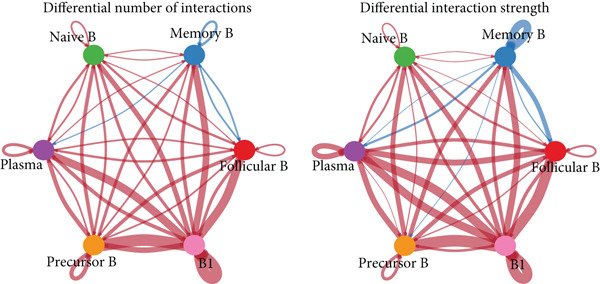
(c)

**Figure:**
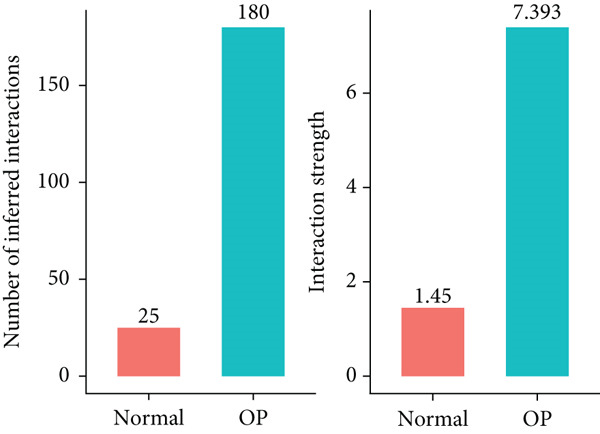
(d)

**Figure:**
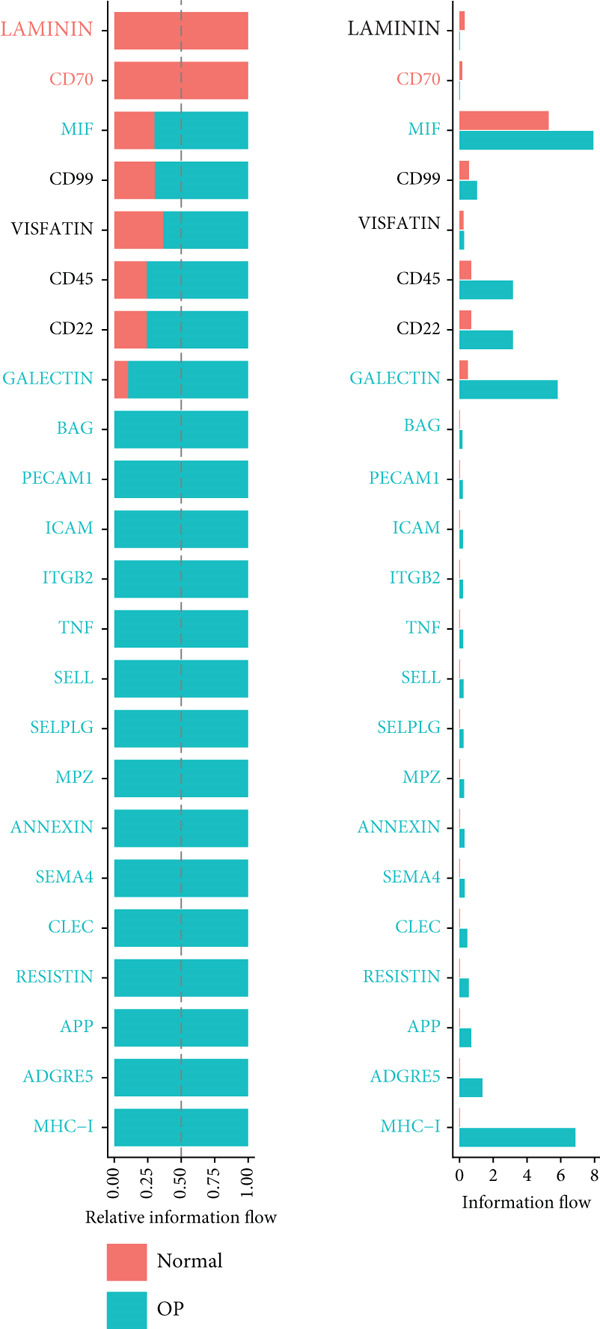
(e)

**Figure:**
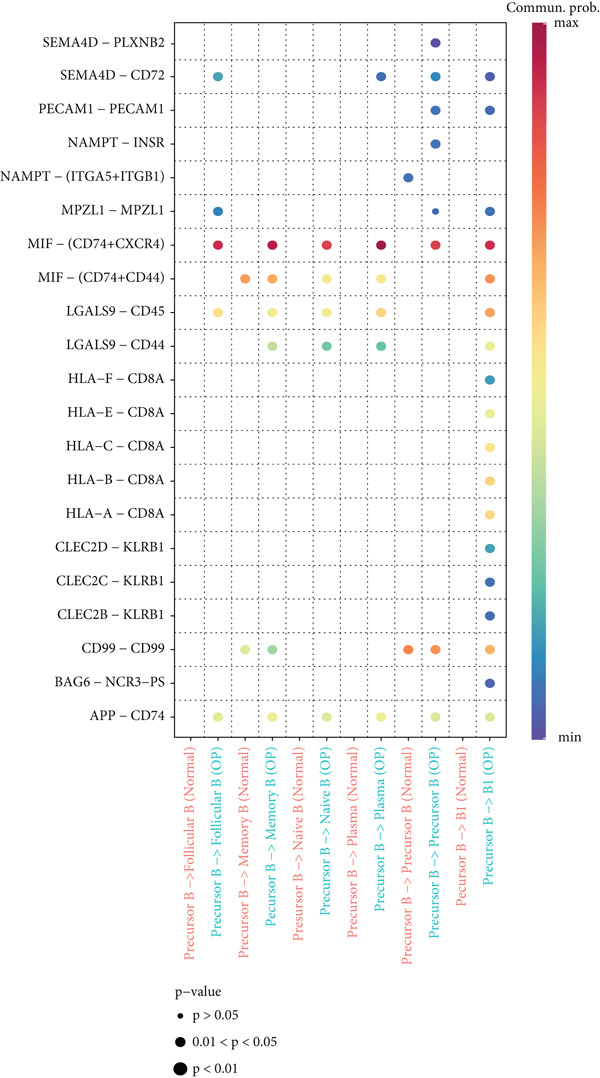
(f)

### 3.6. Analysis of LGALS9‐ and MIF‐Related Pathways

We first focused on the LGALS9‐related pathways. Figure 7a shows that, in the OP group, most LGALS9 pathways were upregulated, except for the pathway between memory B cells with follicular B cells, which was downregulated. And upregulation was particularly evident in precursor B cells and B1 cells. Figure 7b presents the relative contribution of each pathway in this upregulation, where the LGALS9‐CD45 pathway shows the most significant contribution. Figure 7c provides a detailed view of this pathway, emphasizing the changes observed in precursor B cells and follicular B cells. To further investigate, Figure 7d uses a violin plot to analyze the expression changes of ligands in LGALS9‐related pathways across B cell subpopulations. This analysis revealed that LGALS9 expression was increased in precursor B cells and B1 cells, and CD45 and CD44 showed significant upregulation in B1 cells.

Figure 7Analysis of GALECTIN‐ and MIF‐related pathways. (a) Heatmap showing interaction patterns of different B cell subpopulations in the GALECTIN signaling pathway in the normal and OP groups. (b) Relative contribution of receptor‐ligand pairs in the GALECTIN signaling pathway in the OP group. (c) Network diagram illustrating interaction patterns of different B cell subpopulations in the LGALS‐CD45 signaling pathway in the normal and OP groups. (d) Expression differences of GALECTIN‐related pathway molecules in the normal and OP groups. (e) Heatmap showing interaction patterns of different B cell subpopulations in the MIF signaling pathway in the normal and OP groups. (f) Relative contribution of receptor‐ligand pairs in the MIF signaling pathway in the OP group. (g) Network diagram illustrating interaction patterns of different B cell subpopulations in the MIF‐(CD74+CXCR4) signaling pathway in the normal and OP groups. (h) Expression differences of MIF‐related pathway molecules in the normal and OP groups.

**Figure:**
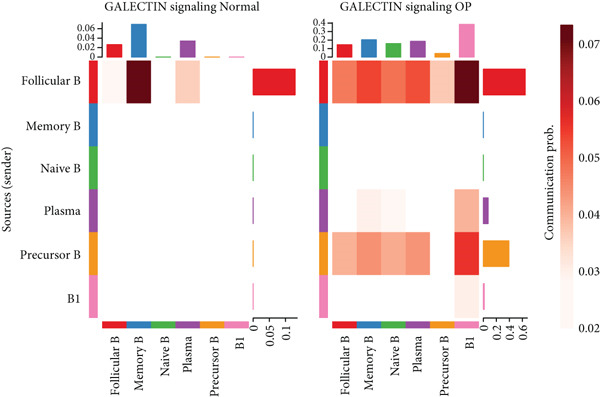
(a)

**Figure:**
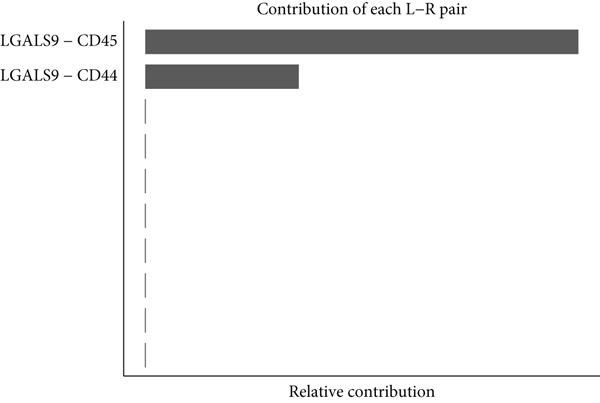
(b)

**Figure:**
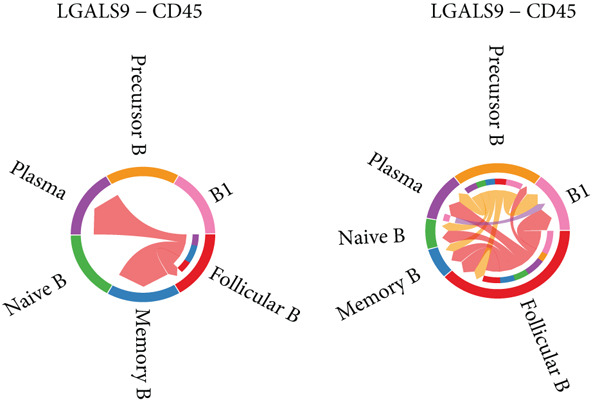
(c)

**Figure:**
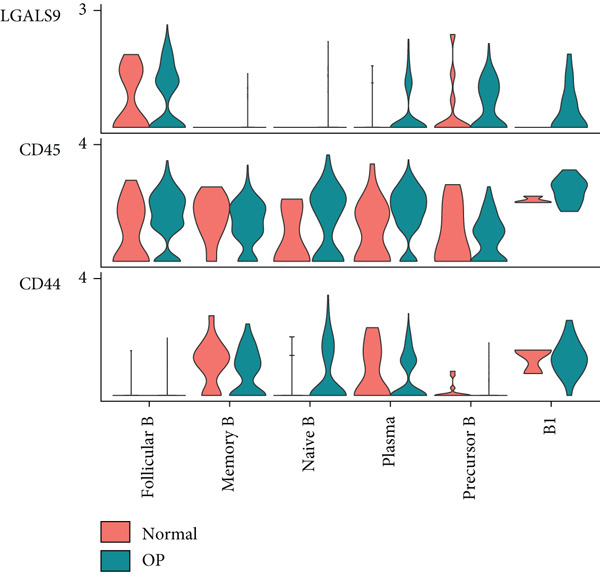
(d)

**Figure:**
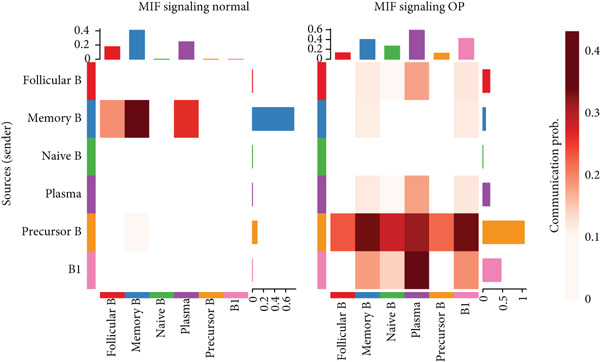
(e)

**Figure:**
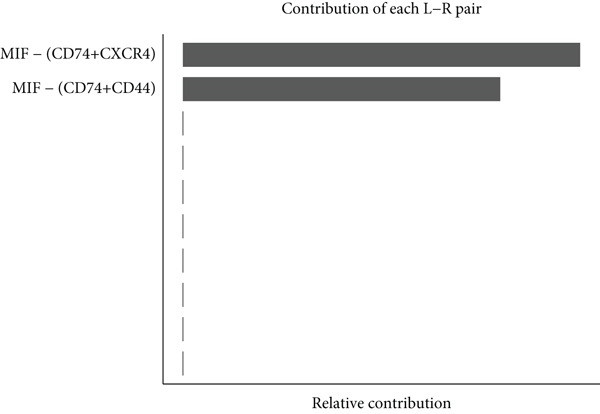
(f)

**Figure:**
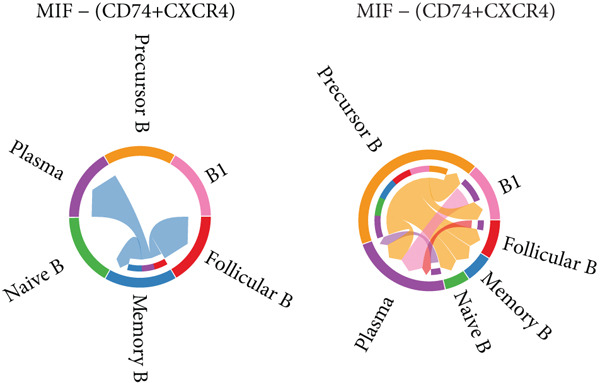
(g)

**Figure:**
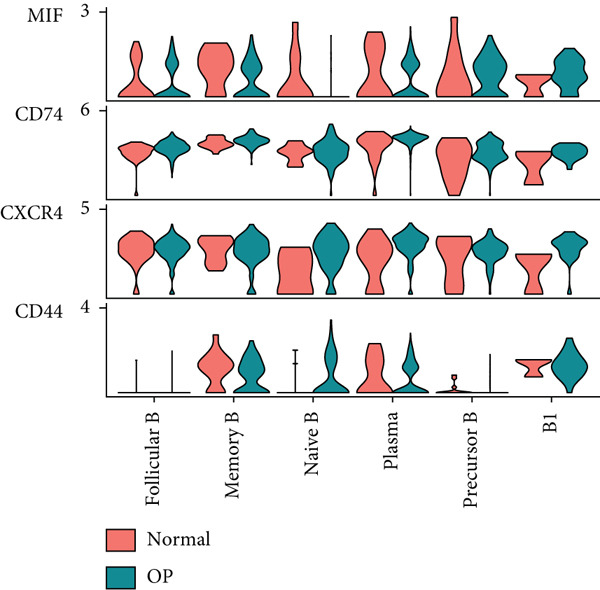
(h)

Next, we analyzed the MIF‐related pathways. Figure 7e shows that most MIF‐related pathways were upregulated in the OP group, with the exception of a few pathways associated with memory B cells. The upregulation was especially significant in pathways related to precursor B cells and B1 cells. Figure 7f displays the relative contribution of each pathway in the MIF‐related signaling, with the MIF‐(CD74+CXCR4) pathway contributing most significantly to this upregulation. Figure 7g provides further insights into the MIF‐(CD74+CXCR4) pathway, focusing on the changes in precursor B cells. Finally, Figure 7h presents a violin plot that analyzes the ligand expression changes in MIF‐related pathways across B cell subpopulations. It shows that in both precursor B cells and B1 cells, the ligands associated with this pathway were upregulated.

## 4. Discussion

In this study, we employed scRNA‐seq analysis to examine the differences in the distribution of specific B cell subpopulations between OP and normal bone tissues. Functional enrichment and differential gene expression analyses revealed that B cells might contribute to the progression of OP through potential pathways associated with viral response and protein folding. Furthermore, our cell–cell communication analysis identified precursor B cells as potential participants in the pathogenesis of OP, with two associated signaling pathways, MIF‐(CD74+CXCR4) and LGALS9‐CD45, possibly playing pivotal roles in this process.

We identified seven major cell populations across four femoral head biopsies, including osteoclasts, osteoblasts, myeloid cells, endothelial cells, T/NK cells, and B cells. Further analysis of B cell distribution revealed six subpopulations: precursor B cells, naïve B cells, follicular B cells, plasma cells, memory B cells, and B1 cells. We found precursor B cells has a higher proportion in OP. The role of precursor B cells in OP has not been traditionally emphasized. A novel study reported that in HIV‐infected individuals, precursor B cells significantly expand with increasing RANKL secretion and reducing OPG. This imbalance in RANKL/OPG ratio enhances osteoclast activity, accelerates bone resorption, and thus leads to reduced bone mineral density (BMD) in the hip, femoral neck, and lumbar spine [28].

Functional enrichment and differential gene analysis revealed that B cells in the OP group were involved in pathways related to viral resistance, likely reflecting immune activation in the disease context [29]. Research has shown that B cells in an inflammatory environment can promote bone resorption and inhibit bone formation through re‐regulation of RANKL and OPG secretion [30]. Additionally, the protein folding pathway was downregulated in the OP group, indicating potential endoplasmic reticulum (ER) stress, which can trigger the unfolded protein response (UPR) [31]. This stress response could impact osteocyte differentiation and function, as ER stress has been shown to inhibit osteoblast differentiation while promoting osteoclast formation [32]. Moreover, B cells in the OP group exhibited downregulation of the nucleic acid‐binding oligomerization domain (NOD) receptor pathway, which is associated with inflammatory activation. Activation of NOD receptors is known to promote the expression of RANKL, fostering osteoclast generation and bone resorption [33, 34].

Cell–cell communication analysis identified two major pathways with upregulated expression among B cell subpopulations: MIF‐(CD74+CXCR4) and LGALS9‐CD45. Mechanistically, MIF and its receptor CD74 play a pivotal role in osteoclast formation by regulating the phosphorylation of NF‐*κ*B‐p65 and ERK1/2, two key signaling molecules involved in osteoclast differentiation and activation [35]. Overexpression of MIF has been linked to high‐turnover OP, a form of bone loss characterized by excessive osteoclast activity and elevated levels of matrix metalloproteinases [36]. And reducing MIF levels was reported to protect against bone loss in OP models of ovariectomy and periodontal disease [37, 38]. Notably, He et al. demonstrated that the small molecule Obacunone can bind to MIF and attenuates RANKL‐induced signaling pathways, including reactive oxygen species, NF‐*κ*B, and MAPK. This attenuation ultimately reduces the expression of the key transcription factor NFATc1 and its downstream osteoclast‐specific proteins [39]. Furthermore, the activation of the MIF‐(CD74+CXCR4) pathway can enhance RANKL expression in B cells by promoting their migration and chemotaxis, affecting osteoclast precursors and dendritic cells, and promoting osteoclast differentiation [40, 41]. In inflammatory conditions, B cells may also enhance RANKL expression in fibroblasts via cytokines such as IL‐1, IL‐6, and TNF‐*α*, thereby contributing to bone resorption [41]. These findings underscore the role of MIF in OP progression and highlight its potential as a therapeutic target.

We also identified the LGALS9‐CD45 pathway. LGALS9 organizes IgM‐BCR into larger clusters, restricting IgM mobility and repositioning inhibitory molecules like CD45 to directly suppress BCR signaling [42]. Although the exact role of this pathway in OP has yet to be fully elucidated, further investigations may provide valuable insights for the development of effective treatment strategies.

This study boasts several key advantages. ScRNA‐seq was used in this study to investigate gene expression and intercellular communication in OP, with a focus on B cell subpopulations. The analysis identified two upregulated pathways, MIF‐(CD74+CXCR4) and LGALS9‐CD45, which may play critical roles in OP progression. Furthermore, the study provides a detailed clustering of B cells, offering new insights into their interactions at different stages of differentiation in OP.

This study has several limitations. The number of available OP samples for scRNA‐seq analysis was insufficient, which may limit the generalizability of the analysis. Larger datasets with more diverse samples are needed to further validate and strengthen the findings. Additionally, the absence of specific markers such as IL‐7R, VpreB, and CD10 in the specimens hindered the further subdivision of precursor B cells. Future studies should address this limitation by incorporating comprehensive marker profiling alongside larger sample sizes. Moreover, the reliability of the results could be further enhanced by integrating more clinical samples and employing multi‐omics approaches.

## 5. Conclusion

This study reveals the heterogeneity of B cells in OP through single‐cell RNA sequencing and provides insights into the cell communication between B cell subpopulations. We identified two upregulated pathways, MIF‐(CD74+CXCR4) and LGALS9‐CD45, that may have essential effects on OP progression. These findings may offer new perspectives in exploring potential targets. Further investigation of these pathways may lead to more effective treatments for OP.

**Nomenclature**
OP: Osteoporosis
BMD: Bone mineral density
RANKL: Receptor activator of nuclear factor‐*κ*B ligand
OPG: Osteoprotegerin
ScRNA‐seq: Single‐cell RNA sequencing
FHTCs: Femoral head tissue cells
UMAP: Uniform manifold approximation and projection
DEGs: Differentially expressed genes
GO: Gene Ontology
KEGG: Kyoto Encyclopedia of Genes and Genomes
MIF: Macrophage migration inhibitory factor
LGALS9: Lectin, galactoside‐binding, soluble, 9
ER: Endoplasmic reticulum
UPR: Unfolded protein response
NOD: Nucleic acid‐binding oligomerization domain
PCA: Principal component analysis

## Ethics Statement

The authors have nothing to report.

## Consent

The authors have nothing to report.

## Conflicts of Interest

The authors declare no conflict of interest.

## Author Contributions

T.Z.: conceptualization, methodology, software, formal analysis, writing—original draft. J.Z.: visualization, writing—review and editing. Y.S. and J.W.: software, writing—review and editing. W.L., Z.Z., J.L., G.F., F.Z.: conceptualization; W.S., G.Z., Y.C.: conceptualization, funding acquisition, resources, supervision. T.Z., J.Z., Z.Z., Y.S., and J.W. contributed equally to this work.

## Funding

This study is supported by the Natural Science Foundation of Guangdong Province (2024A1515012811).

## Data Availability

This study is based on the publicly available dataset GSE169396, which contains single‐cell RNA sequencing data related to osteoporosis. The dataset is accessible at https://www.ncbi.nlm.nih.gov/geo/query/acc.cgi?acc=GSE169396 (accessed on March 24, 2021).
